# Selective psychological benefits of small-group teaching model in junior high school physical education: a randomized pretest–posttest controlled study of physical self-esteem and self-efficacy

**DOI:** 10.3389/fpsyg.2026.1873632

**Published:** 2026-07-08

**Authors:** Deke Liu, Quanhong Lu, Jiawei Shao, Jinluo Axi, Jiangang Qiu, Dongchen Li

**Affiliations:** 1 School of Arts, Chengdu Sport University, Chengdu, China; 2 College of Sports Training, Chengdu Sport University, Chengdu, China; 3 College of Physical Education and Health, Yili Normal University, Yili, China

**Keywords:** physical education, physical self-esteem, physical self-worth, self-efficacy, small-group teaching model

## Abstract

**Introduction:**

Physical education (PE) is a socially evaluative school context in which adolescents’ body-related self-evaluations and efficacy beliefs may be shaped by peer interaction, feedback, and classroom organization. This study examined whether the Small-Group Teaching Model (SGTM) was associated with higher physical self-esteem and general self-efficacy among junior high school students after baseline adjustment, whether its effects were selectively concentrated in physical self-worth, whether gender functioned as a boundary condition, and whether physical self-esteem and self-efficacy showed coordinated change across the intervention period.

**Methods:**

A school-based randomized pretest–posttest controlled design was used. Eighty eighth-grade students from two classes in one middle school in Chengdu, China, participated in a 15-week PE intervention. After baseline assessment, students were individually randomized to either the SGTM condition or a traditional-instruction control condition. The SGTM condition emphasized heterogeneous grouping, task cards, structured peer feedback, and rotating group roles, whereas the control condition followed the same curriculum content and lesson duration under teacher-directed instruction. Physical self-esteem was assessed using the Chinese Physical Self-Esteem Scale, and general self-efficacy was assessed using the Chinese version of the General Self-Efficacy Scale. Data were analyzed using two-way analyses of covariance, tests of homogeneity of regression slopes, Pearson correlations, and Holm correction for multiple comparisons.

**Results:**

After controlling for baseline scores, students in the SGTM condition showed higher posttest self-efficacy and physical self-esteem than those in the control condition. No statistically significant gender or Group × Gender interaction effects were observed for the primary outcomes. At the physical self-perception scale level, physical self-worth was the only scale that remained statistically significant after Holm correction. Physical self-esteem and self-efficacy were positively associated at pretest, posttest, and change-score levels, with the strongest association observed for change scores.

**Discussion:**

These findings suggest that the implemented SGTM instructional package may be associated with more favorable psychological outcomes in PE, particularly general self-efficacy and global physical self-worth, although the effects appeared selective rather than uniformly distributed across domain-specific physical self-perceptions.

## 1 Introduction

Early adolescence is a developmental period characterized by rapid bodily change, intensified social comparison, and substantial reorganization of the self-concept. During this period, body-related self-evaluations are closely linked not only to students’ engagement in physical education (PE), but also to broader indicators of psychological adjustment and well-being (Gualdi-Russo et al., 2022; Sabiston et al., 2019). PE is therefore not only a setting for physical activity, but also a socially evaluative school context in which students’ bodies, abilities, and performances are visible to peers and teachers. Because PE lessons routinely involve observation, comparison, feedback, and judgment, adolescents’ physical self-evaluations and efficacy beliefs should be considered important psychological outcomes of PE instruction rather than incidental by-products of participation (Barker et al., 2023).

The present study focused on physical self-esteem and general self-efficacy because these constructs represent two complementary but distinct psychological resources in PE. Physical self-esteem reflects students’ evaluative relationship with their physical selves, including the extent to which they feel physically competent, valued, and positive about their bodies, and can be understood in relation to the hierarchical model of physical self-perception proposed by Fox and Corbin (1989). By contrast, self-efficacy refers to students’ perceived capability to organize and execute actions required to achieve desired outcomes (Bandura, 1997). Compared with broader self-related constructs such as general self-concept and physical self-concept, physical self-esteem and self-efficacy are more directly connected to the visible, performance-based, and feedback-rich experiences that characterize PE lessons, in which students’ bodies, abilities, and performances are frequently exposed to observation, comparison, and evaluation (Barker et al., 2023; Jankauskienė et al., 2023). In this sense, physical self-esteem captures how students evaluate their physical selves, whereas self-efficacy captures whether students believe they can successfully engage in and complete learning tasks.

The relationship between PE instruction and these psychological outcomes may depend not only on the amount of physical activity provided, but also on how learning tasks, feedback, peer interaction, and responsibility are organized (Casey and Goodyear, 2015; Bechter et al., 2019; Dyson et al., 2022). Traditional teacher-directed instruction can provide clear demonstration, correction, and classroom control. In the present implementation, the traditional teacher-directed condition was designed to emphasize teacher demonstration, individual practice, and teacher feedback, and therefore provided fewer structured opportunities for reciprocal peer feedback, shared responsibility, and student role-taking. Student-centered and group-based pedagogies have attracted increasing attention because they may modify the social and motivational structure of PE lessons (Casey and Goodyear, 2015; Dyson et al., 2022). Importantly, these approaches are not psychologically beneficial by definition; their effects are likely to depend on teaching quality, task design, classroom climate, and fidelity of implementation.

In the present study, the Small-Group Teaching Model (SGTM) is conceptualized as a structured small-group instructional arrangement rather than as a completely novel pedagogical paradigm detached from previous approaches. SGTM shares conceptual features with cooperative learning, reciprocal peer learning, and learner-centered teaching styles, including elements consistent with Mosston and Ashworth (2008) Spectrum of Teaching Styles. In this study, SGTM was operationalized through heterogeneous grouping, shared learning tasks, task cards, structured peer feedback, rotating group roles, and group presentation. Its potential value lies not simply in placing students into groups, but in structuring feedback sources, role responsibility, and peer-supported task engagement during PE lessons.

From a theoretical perspective, SGTM may be linked to self-efficacy through Bandura (1997) framework. Self-efficacy is shaped by mastery experiences, vicarious experiences, social persuasion, and affective states. In PE, a structured small-group setting may offer a relevant theoretical basis for examining students’ efficacy beliefs, as it may reorganize opportunities for task engagement, peer observation, and feedback.

At the same time, the influence of SGTM on physical self-esteem should not be conceptualized as a uniform effect across all body-related domains. According to the hierarchical model of physical self-perceptions proposed by Fox and Corbin (1989), physical self-worth reflects a core and more global evaluative component of physical self-esteem, whereas sport competence, physical condition, body attractiveness, and physical fitness capture more domain-specific physical self-perceptions. This model implies that pedagogical interventions may not affect all components of physical self-perception equally. Based on this hierarchical structure, the expectation that SGTM-related differences may be more evident in physical self-worth than uniformly across domain-specific physical self-perception subscales is presented as a theoretically informed hypothesis rather than as an established effect.

Previous studies and reviews of cooperative learning and student-centered pedagogies in PE have often emphasized participation, motivation, classroom experience, and broad psychosocial outcomes (Casey and Goodyear, 2015; Bechter et al., 2019; Dyson et al., 2022). In Chinese PE contexts, small-group teaching has also been examined in relation to broader psychological and social outcomes. For example, prior studies reported that small-group teaching was associated with psychological health among college or vocational students and with prosocial behavior among college students (Cai, 2006; Fu and Ding, 2008; Zhang, 2010). These studies suggest that small-group teaching has an empirical foundation for examining broader psychological and social outcomes in Chinese PE research. However, most existing studies have focused on broader psychological health, social behavior, participation, or classroom experience, and have been conducted mainly in higher education or vocational education settings. Comparatively less evidence is available regarding whether SGTM is associated with differentiated changes in physical self-esteem, physical self-worth, and domain-specific physical self-perceptions among junior high school students.

Against this background, the present study compared a small-group teaching model with traditional instruction in Grade 8 PE classes, focusing on physical self-esteem, self-efficacy, physical self-worth, and four domain-specific physical self-perception subscales. Rather than asking only whether SGTM is beneficial, the present study sought to identify whether, after baseline adjustment, SGTM is associated with higher physical self-esteem and self-efficacy, whether any psychological benefits are selectively concentrated in particular components of physical self-perception—especially the core physical self-worth scale—and whether gender functions as a meaningful boundary condition for intervention effects. In addition, the study examined whether physical self-esteem and self-efficacy are positively associated not only cross-sectionally, but also at the level of change. Given the single-school context and relatively modest sample size, the present study should be interpreted as providing preliminary school-based evidence rather than definitive evidence of psychological mechanisms.

Based on the literature and theoretical considerations reviewed above, the following hypotheses and research question were formulated:

*H1a*. After controlling for baseline levels, students in the SGTM condition are expected to report higher posttest self-efficacy than students in the traditional-instruction condition.

*H1b*. After controlling for baseline levels, students in the SGTM condition are expected to report higher posttest physical self-esteem than students in the traditional-instruction condition.

*H2*. SGTM-related differences in physical self-perception are expected to show a selective pattern, with evidence anticipated for the core physical self-worth scale rather than uniformly across all domain-specific physical self-perception subscales.

*RQ1*. Does gender function as a potential boundary condition for SGTM-related differences in self-efficacy, physical self-esteem, and physical self-perception scales?

*H3*. Physical self-esteem and self-efficacy are expected to be positively associated at the pretest, posttest, and change-score levels.

## 2 Methods

### 2.1 Design and participants

This study employed a school-based randomized pretest–posttest control-group design. Participants were 80 eighth-grade students recruited from two classes in one middle school in Chengdu, China. The participating school and PE teacher were selected by convenience based on the school’s willingness to participate in the intervention and the availability of two Grade 8 classes with comparable PE schedules. All participants were 13–14 years old. Students were considered eligible if they were able to participate in regular PE classes, had no school-recorded medical contraindications to exercise, and had no parent- or teacher-reported illness or injury that would prevent participation in ordinary PE activities. Biological maturation status was not assessed in the present study.

An *a priori* power analysis was conducted during the study-planning stage using G*Power 3.1 (Faul et al., 2007) to estimate the sample size required for detecting the primary group effect. The initial calculation assumed a medium effect size (Cohen’s *f* = 0.25), an alpha level of 0.05, and statistical power of 0.80. Because of practical constraints in the school-based setting, including the number of eligible Grade 8 classes, class size, scheduling feasibility, and the number of students with parental consent and student assent, the final sample consisted of 80 students. An additional sensitivity analysis indicated that, with the final sample size, the minimum detectable effect size under the present ANCOVA framework was approximately Cohen’s *f* = 0.317 at *α* = 0.05 and power = 0.80. Thus, the study had acceptable sensitivity for detecting medium-to-large effects, whereas statistical power to detect smaller effects, particularly Group × Gender interactions and physical self-perception subscale-level effects, was likely limited.

**Figure 1:**
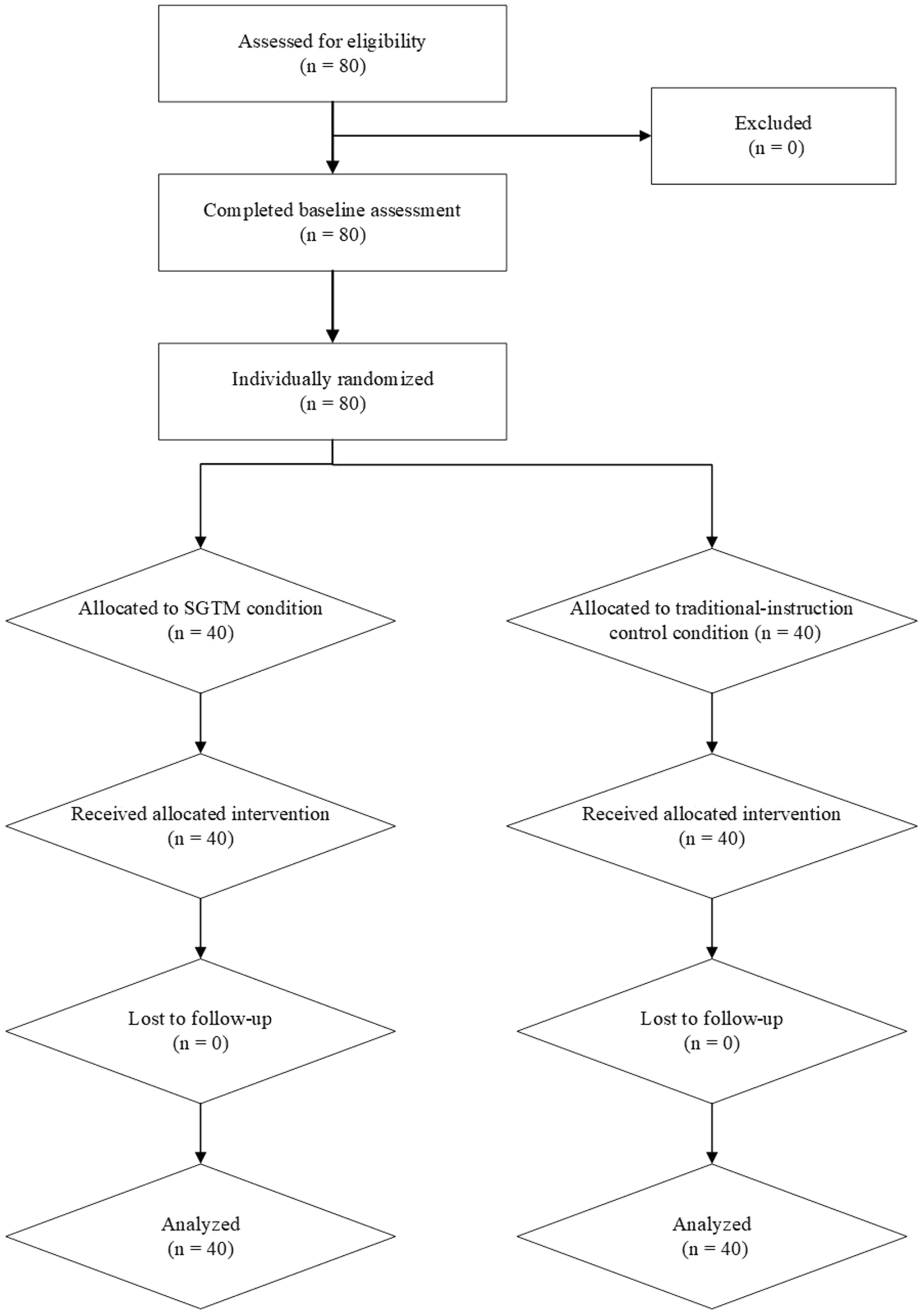
Research process diagram.

After all participants completed the baseline assessment, the 80 eligible students from the two participating classes were pooled and individually randomized using a lot-drawing procedure to either the SGTM condition or the traditional-instruction control condition. Thus, randomization was conducted at the individual student level rather than at the class level. The random allocation procedure was conducted by the research team after baseline assessment, and the PE teacher was not involved in generating the allocation sequence. The experimental group comprised 40 students, including 17 boys and 23 girls, and the control group comprised 40 students, including 18 boys and 22 girls. Descriptive statistics for baseline and posttest outcomes by group and gender are presented in Table 1. A CONSORT-style participant flow diagram is provided in Figure 1.

**Table 1:** Descriptive statistics for self-efficacy and physical self-esteem outcomes by group and gender.

Variable	Time	Total sample (*N* = 80), M ± SD	EG (*n* = 40), M ± SD	CG (*n* = 40), M ± SD	Male (*n* = 35), M ± SD	Female (*n* = 45), M ± SD
Self efficacy	Pretest	28.54 ± 4.47	28.85 ± 5.12	28.23 ± 3.74	29.26 ± 4.37	27.98 ± 4.50
	Posttest	29.79 ± 4.52	30.83 ± 4.88	28.75 ± 3.91	30.69 ± 4.22	29.09 ± 4.67
Physical self-esteem	Pretest	72.90 ± 10.22	74.83 ± 11.37	70.95 ± 8.64	76.29 ± 9.85	70.27 ± 9.82
	Posttest	75.39 ± 9.69	78.50 ± 10.12	72.28 ± 8.24	78.54 ± 8.82	72.82 ± 9.57
Physical self-worth	Pretest	14.69 ± 2.70	15.03 ± 3.14	14.35 ± 2.18	15.09 ± 2.72	14.38 ± 2.68
	Posttest	15.28 ± 2.65	15.95 ± 3.05	14.60 ± 2.01	15.77 ± 2.64	14.89 ± 2.63
Sport competence	Pretest	14.16 ± 2.36	14.58 ± 2.54	13.75 ± 2.12	15.20 ± 1.94	13.36 ± 2.37
	Posttest	14.63 ± 2.31	15.15 ± 2.37	14.10 ± 2.15	15.74 ± 2.01	13.76 ± 2.17
Physical condition	Pretest	14.84 ± 2.09	15.13 ± 2.50	14.55 ± 1.57	15.43 ± 1.85	14.38 ± 2.18
	Posttest	15.33 ± 2.37	15.95 ± 2.74	14.70 ± 1.74	15.94 ± 2.14	14.84 ± 2.44
Body attractiveness	Pretest	14.30 ± 2.58	14.70 ± 3.15	13.90 ± 1.76	14.74 ± 2.52	13.96 ± 2.59
	Posttest	14.74 ± 2.18	15.23 ± 2.56	14.25 ± 1.60	14.94 ± 1.91	14.58 ± 2.37
Physical fitness	Pretest	14.92 ± 3.10	15.40 ± 3.05	14.43 ± 3.11	15.83 ± 2.96	14.20 ± 3.06
	Posttest	15.37 ± 3.03	16.05 ± 2.65	14.68 ± 3.25	16.14 ± 2.65	14.76 ± 3.18

EG, experimental group; CG, control group; M, mean; SD, standard deviation.

### 2.2 Procedure

To reduce teacher-related variability, both the experimental and control groups were instructed by the same experienced PE teacher. The teacher was recruited from the participating school, had 10 years of PE teaching experience, and was not a member of the research team. The teacher was not involved in participant recruitment, questionnaire administration, outcome assessment, or data analysis. The research team coordinated recruitment procedures and administered the pretest and posttest questionnaire assessments using standardized instructions, but did not deliver any PE lessons or participate in students’ classroom evaluation. Before the intervention, the teacher completed a two-week SGTM training program provided by the research team to support consistent implementation of the experimental condition. During the 15-week intervention, the research team conducted unannounced classroom checks every 2 weeks to monitor implementation fidelity, focusing on core SGTM components such as heterogeneous grouping, task-card use, structured peer feedback, and rotating group roles.

After randomization, students received PE instruction according to their assigned condition. The SGTM and control conditions were organized separately to reduce direct contamination between instructional conditions, while the same teacher, curriculum content, lesson duration, and instructional progression were maintained across groups.

The intervention lasted 15 weeks, with four 45-min sessions per week. To ensure a clear and progressive instructional sequence, the curriculum was organized into three phases. Phase 1, from Weeks 1 to 5, focused primarily on the acquisition and familiarization of fundamental motor skills. Phase 2, from Weeks 6 to 10, emphasized skill combinations and their application in task-based practice contexts. Phase 3, from Weeks 11 to 15, shifted toward group competition and integrated physical training, thereby encouraging students to consolidate and apply previously learned skills and fitness components. Each lesson followed the same general structure: an 8-min warm-up, 30 min of skill instruction and practice, and a 7-min cool-down.

The intervention content covered three broad categories: ball-skill learning, strength and power development, and general physical conditioning. The ball-skill module included volleyball and basketball activities, focusing on basic skills such as passing, dribbling, and simple cooperative play. The strength and power module included standing long jump and pull-ups, targeting lower-limb explosive power and upper-body strength, respectively. The general conditioning module primarily involved rope skipping, which was used to develop coordination, rhythm control, and basic physical fitness. Across the 15-week period, the curriculum progressed from basic skill acquisition to combined practice and then to integrated application.

Students in the experimental group received instruction under the SGTM. Within the SGTM condition, students were organized into heterogeneous groups primarily according to observed fitness and skill levels. Social familiarity was considered only as a secondary practical factor to facilitate communication and reduce initial discomfort within groups. Each group consisted of five students, resulting in eight groups in total. The instructional sequence in the experimental condition consisted of teacher demonstration, collaborative group practice, and group presentation. During the practice phase, task cards were used to facilitate structured interaction and shared responsibility. Students who were not actively performing were required to record their peers’ success rates and provide at least one concrete suggestion for improvement, thereby creating a built-in mechanism for peer evaluation and mutual assistance. In addition, each group had one student leader who was responsible for coordinating task completion and group feedback. This leadership role rotated weekly so that all students had opportunities to assume organizational responsibility and develop a stronger sense of autonomy and engagement. In the experimental group, all activities, including volleyball, basketball, standing long jump, pull-ups, and rope skipping, were implemented through cooperative group tasks, structured peer feedback, and rotating role assignments.

The control group followed the same curriculum content, instructional progression, lesson duration, and scheduled practice time as the experimental group. The instructional sequence in the control condition was teacher demonstration, individual practice, teacher-guided rotation, and random group practice. Although incidental communication among students was permitted, no structured peer-assistance tasks were assigned and no explicit team goals were emphasized. Feedback was provided directly by the teacher to individual students rather than being mediated through peer interaction. Thus, the critical distinction between the two conditions lay not in lesson content or practice duration, but in the structure of classroom interaction, the source of feedback, and the extent to which students assumed active roles in the learning process.

### 2.3 Measures

#### 2.3.1 Physical self-esteem

Physical self-esteem was assessed using the Chinese revised Physical Self-Esteem Scale by Xu and Yao (2001). The instrument contains one core scale, physical self-worth, and four domain-specific physical self-perception subscales: sport competence, physical condition, body attractiveness, and physical fitness. Physical self-worth reflects a more global evaluative judgment of the physical self, whereas the four domain-specific subscales capture more specific perceptions of physical capability, physical condition, appearance, and fitness.

The instrument contains 30 items, with six items for each scale, rated on a 4-point scale. Scores for each scale range from 6 to 24, and higher scores indicate more positive physical self-perceptions. Following the scoring procedure of the revised scale, an overall physical self-esteem score was calculated by summing the five scale scores, with total scores ranging from 30 to 120. In the present sample, Cronbach’s *α* was 0.804 for the physical self-esteem scale. The Cronbach’s *α* values for the five physical self-perception scales were 0.801 for physical self-worth, 0.796 for sport competence, 0.857 for physical condition, 0.790 for body attractiveness, and 0.845 for physical fitness.

#### 2.3.2 Self-efficacy

Self-efficacy was measured using the Chinese version of the General Self-Efficacy Scale revised and validated by Wang et al. (2001). The scale contains 10 items, each rated on a 4-point scale ranging from 1 = strongly disagree to 4 = strongly agree. Total scores range from 10 to 40, with higher scores indicating higher levels of general self-efficacy. The Chinese version of the scale has demonstrated acceptable reliability and validity in previous validation work. In the present sample, Cronbach’s *α* for the scale was 0.849.

### 2.4 Data collection

Questionnaires were administered 1 week before the intervention and 1 week after the intervention by trained members of the research team using standardized instructions. The PE teacher was not involved in questionnaire administration and did not have access to individual questionnaire responses. Students were informed that participation was voluntary, that there were no right or wrong answers, and that their responses would not affect their PE grades or classroom evaluation. All students completed the questionnaires anonymously and returned them immediately on site. The response rate was 100% at both measurement occasions, and all randomized students were included in the final analyses.

### 2.5 Statistical analysis

All statistical analyses were performed using IBM SPSS Statistics 27.0. Descriptive statistics were first computed for each variable and are presented as means and standard deviations (M ± SD). The physical self-esteem score was calculated as the sum of physical self-worth and the four domain-specific physical self-perception subscales: sport competence, physical condition, body attractiveness, and physical fitness.

For the primary intervention analyses, two-way analyses of covariance (ANCOVAs) were conducted. Posttest scores were used as the dependent variables, corresponding pretest scores were entered as covariates, and group and gender were included as fixed factors. The group effect was used to test the primary intervention hypotheses, whereas the main effect of gender and the Group × Gender interaction were examined to address the research question concerning gender as a possible boundary condition. Before interpreting the ANCOVA results, the homogeneity of regression slopes assumption was tested. The ANCOVA results were interpreted when no significant covariate-by-factor interaction was detected.

The ANCOVA results are reported using *F* values, *p* values, and partial eta squared (*ηp*^2^). Adjusted mean differences with 95% confidence intervals were reported for group and gender main-effect comparisons, whereas Group × Gender interactions were reported using *F* values, *p* values, and *ηp*^2^. To control for Type I error accumulation caused by multiple comparisons, Holm correction was applied separately to the family of tests for physical self-worth and the four domain-specific physical self-perception subscales. Specifically, Holm correction was applied separately to the group effects, gender effects, and Group × Gender interaction effects.

Pearson correlation analyses were used to examine associations between self-efficacy and physical self-esteem at the pretest, posttest, and change-score levels. Pearson correlation coefficients were reported with 95% confidence intervals. Change scores were calculated as posttest score minus pretest score. Additional correlation analyses were conducted between self-efficacy and physical self-worth, sport competence, physical condition, body attractiveness, and physical fitness. Holm correction was applied to subscale-level correlation analyses. Change-score correlations were interpreted as evidence of covariation in change rather than causal or directional associations. The statistical significance level was set at *p* < 0.05.

### 2.6 Ethics statement

All procedures in this study were conducted in accordance with the relevant institutional guidelines and ethical standards for research involving human participants. The study protocol was reviewed and approved by the Ethics Committee of Chengdu Sport University (Approval No.: Ethics Committee of Chengdu Sport University [2025] No. 191).

### 2.7 Informed consent

Written informed consent was obtained from students’ parents or legal guardians, and assent was obtained from all participating students. Participation was voluntary, and students were informed that they could withdraw at any time without academic or PE-related consequences. All questionnaire responses were anonymous and used only for research purposes.

## 3 Results

### 3.1 Descriptive statistics

Descriptive statistics for self-efficacy, physical self-esteem, physical self-worth, and the four domain-specific physical self-perception subscales are presented in Tables 1, 2. Descriptively, posttest scores for self-efficacy and physical self-esteem were higher in the SGTM group than in the control group. Specifically, the SGTM group reported a posttest self-efficacy score of 30.83 ± 4.88, compared with 28.75 ± 3.91 in the control group. Similarly, the posttest physical self-esteem score was 78.50 ± 10.12 in the SGTM group and 72.28 ± 8.24 in the control group. Across the full sample, self-efficacy increased from 28.54 ± 4.47 at pretest to 29.79 ± 4.52 at posttest, while physical self-esteem increased from 72.90 ± 10.22 to 75.39 ± 9.69. Descriptive statistics stratified by gender and group are presented in Table 2.

**Table 2:** Pretest and posttest descriptive statistics by group and gender.

Variable	Gender	Group	Pretest M ± SD	Posttest M ± SD
Physical self-worth	Male	CG (*n* = 18)	14.72 ± 2.27	14.78 ± 2.01
		EG (*n* = 17)	15.47 ± 3.15	16.82 ± 3.05
	Female	CG (*n* = 22)	14.05 ± 2.10	14.46 ± 2.24
		EG (*n* = 23)	14.70 ± 3.15	15.30 ± 2.95
Sport competence	Male	CG (*n* = 18)	15.06 ± 1.95	15.33 ± 1.75
		EG (*n* = 17)	15.35 ± 1.97	16.18 ± 2.22
	Female	CG (*n* = 22)	12.68 ± 1.62	13.09 ± 1.93
		EG (*n* = 23)	14.00 ± 2.80	14.39 ± 2.23
Physical condition	Male	CG (*n* = 18)	15.22 ± 1.35	15.28 ± 1.53
		EG (*n* = 17)	15.65 ± 2.29	16.65 ± 2.49
	Female	CG (*n* = 22)	14.00 ± 1.54	14.23 ± 1.98
		EG (*n* = 23)	14.74 ± 2.23	15.43 ± 2.84
Body attractiveness	Male	CG (*n* = 18)	14.39 ± 1.24	14.72 ± 1.02
		EG (*n* = 17)	15.12 ± 3.41	15.18 ± 2.56
	Female	CG (*n* = 22)	13.50 ± 2.09	13.86 ± 1.89
		EG (*n* = 23)	14.39 ± 2.98	15.26 ± 2.62
Physical fitness	Male	CG (*n* = 18)	15.61 ± 3.20	15.89 ± 2.89
		EG (*n* = 17)	16.06 ± 2.75	16.41 ± 2.43
	Female	CG (*n* = 22)	13.46 ± 2.74	13.68 ± 3.24
		EG (*n* = 23)	14.91 ± 3.23	15.78 ± 2.83
Physical self-esteem	Male	CG (*n* = 18)	74.95 ± 7.73	75.78 ± 6.67
		EG (*n* = 17)	77.65 ± 11.77	81.29 ± 10.29
	Female	CG (*n* = 22)	67.68 ± 8.08	69.41 ± 8.40
		EG (*n* = 23)	72.74 ± 10.85	76.44 ± 9.70
Self-efficacy	Male	CG (*n* = 18)	29.39 ± 3.20	30.17 ± 3.29
		EG (*n* = 17)	29.17 ± 5.45	31.24 ± 5.07
	Female	CG (*n* = 22)	27.27 ± 3.95	27.59 ± 4.07
		EG (*n* = 23)	28.65 ± 4.97	30.52 ± 4.83

EG, experimental group; CG, control group; M, mean; SD, standard deviation.

### 3.2 Test for homogeneity of regression slopes

Before conducting the ANCOVA analyses, the homogeneity of regression slopes assumption was examined. For self-efficacy, physical self-esteem, physical self-worth, and the four domain-specific physical self-perception subscales, the covariate-by-factor interaction terms were not statistically significant, including the interactions between pretest scores and group, pretest scores and gender, and pretest scores and the Group × Gender term. These results supported the homogeneity of regression slopes assumption for ANCOVA. Detailed results are presented in Table 3.

**Table 3:** Homogeneity of regression slopes tests for self-efficacy and physical self-esteem outcomes.

Dependent variable × effect	*F*	*p*	*ηp* ^2^
Gender × pretest physical self-esteem	0.726	0.397	0.010
Group × pretest physical self-esteem	0.144	0.705	0.002
Gender × group × pretest physical self-esteem	0.834	0.364	0.011
Gender × pretest self-efficacy	1.019	0.316	0.014
Group × pretest self-efficacy	0.478	0.491	0.007
Gender × group × pretest self-efficacy	0.476	0.492	0.007
Group × physical self-worth	0.507	0.479	0.007
Group × sport competence	0.124	0.726	0.002
Group × physical condition	0.015	0.902	0.000
Group × body attractiveness	1.311	0.256	0.018
Group × physical fitness	3.080	0.084	0.041
Gender × physical self-worth	0.761	0.386	0.010
Gender × sport competence	0.320	0.573	0.004
Gender × physical condition	0.010	0.920	0.000
Gender × body attractiveness	0.351	0.556	0.005
Gender × physical fitness	1.847	0.178	0.025
Group × gender × physical self-worth	0.974	0.327	0.013
Group × gender × sport competence	1.321	0.254	0.018
Group × gender × physical condition	0.285	0.595	0.004
Group × gender × body attractiveness	0.020	0.889	0.000
Group × gender × physical fitness	0.272	0.604	0.004

*ηp*^2^, partial eta squared.

### 3.3 Effects of SGTM on self-efficacy and physical self-esteem

After controlling for baseline scores, the two-way ANCOVA showed a statistically significant group effect on self-efficacy, *F* = 13.610, *p* < 0.001, *ηp*^2^ = 0.154. Students in the SGTM condition showed higher posttest self-efficacy than students in the traditional-instruction condition, with an adjusted mean difference of 1.505, 95% CI [0.692, 2.318]. The main effect of gender was not statistically significant, *F* = 1.433, *p* = 0.235, *ηp*^2^ = 0.019. The Group × Gender interaction was also not statistically significant, *F* = 0.226, *p* = 0.636, *ηp*^2^ = 0.003.

For physical self-esteem, the group effect was also statistically significant, *F* = 8.973, *p* = 0.004, *ηp*^2^ = 0.107. Students in the SGTM condition showed higher posttest physical self-esteem than students in the traditional-instruction condition, with an adjusted mean difference of 3.260, 95% CI [1.092, 5.428]. The main effect of gender was not statistically significant, *F* = 0.635, *p* = 0.428, *ηp*^2^ = 0.008. The Group × Gender interaction was likewise not statistically significant, *F* = 0.022, *p* = 0.882, *ηp*^2^ = 0.000.

These findings were consistent with H1a and H1b, indicating that students in the SGTM condition showed higher posttest self-efficacy and physical self-esteem than students in the traditional-instruction condition after baseline adjustment. With respect to RQ1, no statistically significant Group × Gender interaction was observed for either primary outcome. Given the modest sample size and limited statistical power for detecting interaction effects, these non-significant interaction findings should be interpreted cautiously and should not be taken as definitive evidence that gender did not influence students’ responses to the intervention. Detailed ANCOVA results, including adjusted mean differences and 95% confidence intervals for group and gender main-effect comparisons, are presented in Table 4.

**Table 4:** ANCOVA results for self-efficacy and physical self-esteem.

Dependent variable	Effect	Level	Adjusted mean	Adjusted mean difference	95% CI	*F*	*p*	*ηp* ^2^
Self-efficacy	Group	EG	30.568	1.505	[0.692, 2.318]	13.610	<0.001	0.154
		CG	29.063					
	Gender	Male	30.062	0.493	[−0.327, 1.313]	1.433	0.235	0.019
		Female	29.570					
	Group* Gender	EG male	30.718	—	—	0.226	0.636	0.003
		EG female	30.419					
		CG male	29.407					
		CG female	28.720					
Physical Self-esteem	Group	EG	77.075	3.260	[1.092, 5.428]	8.973	0.004	0.107
		CG	73.815					
	Gender	Male	75.891	0.892	[−1.338, 3.122]	0.635	0.428	0.008
		Female	74.999					
	Group* Gender	EG male	77.601	—	—	0.022	0.882	0.000
		EG female	76.550					
		CG male	74.182					
		CG female	73.449					

EG, experimental group; CG, control group; CI, confidence interval; *ηp*^2^, partial eta squared. Adjusted mean differences are presented as EG − CG for group effects and male − female for gender effects. Confidence intervals are reported for group and gender main-effect comparisons. Group × Gender interactions are reported using F, *p*, and *ηp*^2^.

### 3.4 Effects on physical self-worth and domain-specific physical self-perception subscales

Further ANCOVAs were conducted for physical self-worth and the four domain-specific physical self-perception subscales. Before Holm correction, statistically significant group effects were observed for physical self-worth, *F* = 7.768, *p* = 0.007, *ηp*^2^ = 0.094, and physical condition, *F* = 5.280, *p* = 0.024, *ηp*^2^ = 0.066, whereas group effects for sport competence, body attractiveness, and physical fitness were not statistically significant. For gender, only sport competence showed a statistically significant uncorrected main effect, *F* = 4.958, *p* = 0.029, *ηp*^2^ = 0.062. No statistically significant Group × Gender interaction was observed for any physical self-perception scale.

Because these analyses involved multiple comparisons, Holm-adjusted *p* values were examined separately for group effects, gender effects, and Group × Gender interaction effects. After Holm correction, only the group effect for physical self-worth remained statistically significant, adjusted mean difference = 0.897, 95% CI [0.256, 1.539], Holm-adjusted *p* = 0.035. The group effect for physical condition no longer reached statistical significance, Holm-adjusted *p* = 0.096, and the uncorrected gender effect for sport competence also did not remain statistically significant, Holm-adjusted *p* = 0.145. The Group × Gender interaction effects also remained non-significant after correction.

These findings were consistent with H2 in that physical self-worth was the only physical self-perception scale that retained a statistically significant group effect after Holm correction. However, because no direct statistical comparison of effect sizes across subscales was conducted, this pattern should not be interpreted as evidence that the effect on physical self-worth was statistically stronger than the effects on the other physical self-perception scales. The absence of statistically significant Group × Gender interactions should also be interpreted cautiously, given the modest sample size and limited statistical power for detecting interaction effects. Detailed ANCOVA results, including adjusted mean differences and 95% confidence intervals for group and gender main-effect comparisons, are presented in Table 5.

**Table 5:** Formal test results for the five subdimensions.

Dimension	Effect	Level 1 adjusted mean	Level 2 adjusted mean	Adjusted mean difference	95% CI	*F*	*p*	*ηp* ^2^	Holm- adjusted *p*
Physical self-worth	Group	15.753	14.855	0.897	[0.256, 1.539]	7.768	0.007	0.094	0.035
	Gender	Male 15.479	Female 15.129	0.350	[−0.292, 0.992]	1.179	0.281	0.015	1.000
	Group* Gender	EG male 16.207; EG female 15.298	CG male 14.750; CG female 14.960	—	—	3.070	0.084	0.039	0.420
Sport competence	Group	14.962	14.396	0.566	[−0.141, 1.273]	2.547	0.115	0.033	0.345
	Gender	Male 15.103	Female 14.255	0.848	[0.089, 1.607]	4.958	0.029	0.062	0.145
	Group* Gender	EG male 15.432; EG female 14.493	CG male 14.775; CG female 14.017	—	—	0.067	0.797	0.001	1.000
Physical condition	Group	15.762	14.930	0.831	[0.111, 1.552]	5.280	0.024	0.066	0.096
	Gender	Male 15.493	Female 15.199	0.295	[−0.443, 1.032]	0.633	0.429	0.008	1.000
	Group* Gender	EG male 16.011; EG female 15.512	CG male 14.976; CG female 14.885	—	—	0.325	0.570	0.004	1.000
Body attractiveness	Group	14.926	14.522	0.403	[−0.217, 1.024]	1.677	0.199	0.022	0.345
	Gender	Male 14.657	Female 14.791	−0.134	[−0.754, 0.487]	0.184	0.669	0.002	1.000
	Group* Gender	EG male 14.649; EG female 15.202	CG male 14.665; CG female 14.379	—	—	1.855	0.177	0.024	0.708
Physical fitness	Group	15.659	15.075	0.583	[−0.220, 1.387]	2.092	0.152	0.027	0.345
	Gender	Male 15.445	Female 15.289	0.156	[−0.668, 0.980]	0.143	0.707	0.002	1.000
	Group* Gender	EG male 15.536; EG female 15.782	CG male 15.355; CG female 14.796	—	—	1.015	0.317	0.013	0.951

EG, experimental group; CG, control group; CI, confidence interval; *ηp*^2^, partial eta squared. For group effects, adjusted mean differences are presented as EG − CG. For gender effects, adjusted mean differences are presented as male − female. Confidence intervals are reported for group and gender main-effect comparisons. Group × Gender interactions are reported using *F*, *p*, *ηp*^2^, and Holm-adjusted *p*. Holm-adjusted *p*-values were applied separately to each family of tests.

### 3.5 Correlations between physical self-esteem and self-efficacy

Pearson correlation analyses showed that physical self-esteem and self-efficacy were positively associated at the pretest, posttest, and change-score levels. Pretest physical self-esteem was significantly correlated with pretest self-efficacy, *r* = 0.451, 95% CI [0.257, 0.610], *p* < 0.001. Posttest physical self-esteem was also significantly correlated with posttest self-efficacy, *r* = 0.544, 95% CI [0.368, 0.682], *p* < 0.001. The largest association was observed between change in physical self-esteem and change in self-efficacy, *r* = 0.743, 95% CI [0.625, 0.828], *p* < 0.001.

These results were consistent with H3, indicating that physical self-esteem and self-efficacy were positively associated across baseline, posttest, and change-score levels. Students who showed greater increases in physical self-esteem also tended to show greater increases in self-efficacy. However, the change-score association should be interpreted as covariation in change rather than evidence of a causal or directional relationship. Detailed correlation results are presented in Table 6.

**Table 6:** Correlations between physical self-esteem and self-efficacy.

Variables	*r*	95% CI	*p*	*N*
Pretest physical self-esteem and pretest self-efficacy	0.451	[0.257, 0.610]	<0.001	80
Posttest physical self-esteem and posttest self-efficacy	0.544	[0.368, 0.682]	<0.001	80
Change in physical self-esteem and change in self-efficacy	0.743	[0.625, 0.828]	<0.001	80

CI, confidence interval. Change scores were calculated as posttest score minus pretest score.

### 3.6 Correlations between physical self-perception scales and self-efficacy

Additional Pearson correlation analyses examined the associations between self-efficacy and physical self-worth, sport competence, physical condition, body attractiveness, and physical fitness at the pretest, posttest, and change-score levels. At pretest, all five physical self-perception scales were positively associated with self-efficacy, with correlation coefficients ranging from *r* = 0.226, 95% CI [0.007, 0.425], to *r* = 0.436, 95% CI [0.239, 0.598]. At posttest, the correlations ranged from *r* = 0.299, 95% CI [0.085, 0.487], to *r* = 0.503, 95% CI [0.318, 0.651]. At the change-score level, the correlations ranged from *r* = 0.332, 95% CI [0.121, 0.514], to *r* = 0.579, 95% CI [0.412, 0.709].

The largest change-score correlations were observed for physical self-worth, *r* = 0.579, 95% CI [0.412, 0.709], *p* < 0.001, and sport competence, *r* = 0.576, 95% CI [0.408, 0.706], *p* < 0.001. After Holm correction, all tested correlations remained statistically significant, although the pretest association between physical fitness and self-efficacy was relatively small, *r* = 0.226, 95% CI [0.007, 0.425], Holm-adjusted *p* = 0.044. Overall, these findings suggest stable positive associations between self-efficacy and physical self-perception scales across pretest, posttest, and change-score levels. These associations should be interpreted as correlational patterns rather than evidence of causal or directional relationships. Detailed correlation results are presented in Table 7.

**Table 7:** Correlations between the five physical self-perception scales and self-efficacy at pretest, posttest, and change-score levels.

Dimension	Pretest *r* [95% CI]	Pretest *p*	Posttest *r* [95% CI]	Posttest *p*	Change-score *r* [95% CI]	Change-score *p*	Holm-adjusted *p* (Pretest)	Holm-adjusted *p* (Posttest)	Holm-adjusted *p* (Change)
Physical self-worth	0.436 [0.239, 0.598]	<0.001	0.499 [0.314, 0.648]	<0.001	0.579 [0.412, 0.709]	<0.001	0.005	0.005	0.005
Sport competence	0.412 [0.211, 0.579]	<0.001	0.503 [0.318, 0.651]	<0.001	0.576 [0.408, 0.706]	<0.001	0.005	0.005	0.005
Physical condition	0.353 [0.144, 0.531]	0.001	0.443 [0.247, 0.604]	<0.001	0.461 [0.268, 0.618]	<0.001	0.005	0.005	0.005
Body attractiveness	0.395 [0.192, 0.566]	<0.001	0.395 [0.192, 0.566]	<0.001	0.364 [0.157, 0.540]	<0.001	0.005	0.005	0.005
Physical fitness	0.226 [0.007, 0.425]	0.044	0.299 [0.085, 0.487]	0.007	0.332 [0.121, 0.514]	0.003	0.044	0.007	0.005

CI, confidence interval. Change scores were calculated as posttest score minus pretest score. Holm-adjusted *p*-values were applied separately to pretest, posttest, and change-score correlations.

## 4 Discussion

### 4.1 Main effects

This study examined whether students in a structured SGTM condition showed higher posttest self-efficacy and physical self-esteem than those in a traditional-instruction condition after controlling for baseline levels. The results showed statistically significant group effects for both outcomes and were consistent with H1a and H1b. Students in the SGTM condition showed higher posttest self-efficacy and physical self-esteem than those in the traditional-instruction condition. These findings suggest that the potential psychological value of SGTM may lie not only in changing classroom organization, but also in creating a socio-motivational learning context characterized by structured peer interaction, shared responsibility, and active participation. However, the present study did not directly measure the psychological mechanisms underlying these effects; therefore, the following explanations should be interpreted as theoretically plausible rather than definitive.

The finding for self-efficacy is consistent with Bandura’s (1997) framework, according to which efficacy beliefs are shaped by mastery experiences, vicarious experiences, and social persuasion. In the present intervention, heterogeneous grouping, task cards, rotating roles, and structured peer feedback may have provided learning conditions consistent with these efficacy-building sources. Compared with traditional teacher-directed instruction, the SGTM condition may have offered students more opportunities to experience manageable task success, observe peers with similar ability levels, and receive immediate encouragement or corrective feedback in a smaller-group setting. These features may help explain why students in the SGTM condition reported higher posttest self-efficacy. Nevertheless, because these proposed processes were not directly assessed, this interpretation remains speculative and should be tested in future studies using process measures or mixed-methods designs.

The finding for physical self-esteem also suggests that the implemented SGTM instructional package may have been associated with more favorable body-related psychological outcomes. PE is a visible and socially evaluative context in which students’ bodies, abilities, and performance are often exposed to peer comparison. A classroom structure involving cooperative tasks, mutual assistance, rotating roles, and group-based participation may reduce the dominance of public comparison and provide more opportunities for students to experience competence and recognition. This interpretation is consistent with prior work showing that student-centered learning strategies in PE can support autonomous motivation, learning efficacy, and need satisfaction (Bechter et al., 2019), and with evidence linking supportive PE environments to more positive body-related psychological outcomes (Cox et al., 2017; Jankauskienė et al., 2023). Thus, the observed benefits should be interpreted as being associated with the implemented SGTM instructional package—including grouping structure, task cards, peer feedback, rotating roles, and teacher guidance—rather than as evidence that SGTM as a label alone caused the outcomes.

### 4.2 Selective effects on physical self-worth

A notable finding of this study was that the group effect was not uniformly observed across all physical self-perception scales. Before multiple-comparison correction, group effects were observed for physical self-worth and physical condition. However, after Holm correction, only the group effect for physical self-worth remained statistically significant. This pattern was consistent with H2, but it should be interpreted cautiously because no direct statistical comparison of effect sizes across subscales was conducted. Therefore, the finding should not be taken as evidence that the effect on physical self-worth was statistically stronger than the effects on the other physical self-perception scales.

This pattern can be understood in relation to the hierarchical structure of physical self-perception. In the framework proposed by Fox and Corbin (1989), physical self-worth represents a more global evaluative component of the physical self, whereas sport competence, physical condition, body attractiveness, and physical fitness reflect more domain-specific perceptions. This corrected pattern is consistent with this hierarchical framework. It may suggest that the implemented SGTM instructional package was more closely associated with students’ general evaluative relationship with their physical selves than with consistent changes across all specific physical domains.

One possible explanation is that SGTM may have modified the social and evaluative climate of the PE lesson. Through structured peer support, shared responsibility, rotating roles, and reduced reliance on teacher-only evaluation, students may have experienced their participation as more valued and less dependent on public comparison or performance ranking. Such classroom conditions may be particularly relevant to physical self-worth because this component reflects a broader sense of physical value and acceptance. However, this interpretation remains theoretical, as the present study did not directly measure perceived evaluative threat, peer support quality, classroom climate, or students’ subjective sense of recognition.

By contrast, more domain-specific perceptions, such as sport competence, body attractiveness, and physical fitness, may require longer exposure, more intensive skill development, measurable changes in physical performance, or changes in body-related experiences beyond PE lessons. Therefore, the absence of statistically significant corrected effects across all domain-specific subscales should not be interpreted as intervention failure. Rather, the present findings suggest a selective pattern centered on the more global evaluative component of physical self-perception. Future studies should directly assess potential mediating processes and compare effect patterns across physical self-perception domains using larger samples and longer follow-up periods.

### 4.3 Gender as a boundary condition

The present study also examined whether gender functioned as a potential boundary condition for students’ responses to the SGTM intervention. In response to RQ1, neither the main effect of gender nor the Group × Gender interaction was statistically significant for the primary outcomes. At the physical self-perception scale level, the uncorrected gender effect for sport competence did not remain statistically significant after Holm correction, and no Group × Gender interaction was statistically significant. These findings suggest that, within the present sample and classroom context, there was no clear evidence that students’ responses to the SGTM condition differed by gender. However, this should not be interpreted as definitive evidence that gender did not influence the intervention response.

This result should be interpreted cautiously. It does not imply that gender is unimportant in PE-related psychological development. Previous research has consistently shown gender differences in physical self-concept, body-related concerns, and experiences of sport and PE (Klomsten et al., 2004; Klomsten et al., 2005; Slater and Tiggemann, 2011). However, mean-level gender differences and gender as an intervention boundary condition are conceptually distinct. Students may differ by gender in average levels of a psychological construct without necessarily showing different responsiveness to a specific instructional condition. Therefore, the present findings should be understood as indicating that statistically significant gender moderation was not detected in this sample, rather than as evidence that gender is irrelevant to SGTM-related outcomes.

Another important consideration is statistical power. Although the total sample provided acceptable sensitivity for detecting medium-to-large group effects, the study had limited sensitivity for smaller interaction effects. Once students were divided by both group and gender, the cell sizes were modest. Therefore, the absence of statistically significant Group × Gender interactions should not be read as strong evidence against the possibility of gender moderation in SGTM-related psychological outcomes. Future studies with larger and more gender-balanced samples should examine whether gender-related differences emerge under different PE content, grouping strategies, teacher practices, or classroom climates. This is especially important because gendered experiences in PE may be shaped not only by biological sex, but also by perceived competence, peer norms, body-related anxiety, and motivational quality (Haug et al., 2023; Jankauskienė et al., 2023).

### 4.4 Coordinated change between physical self-esteem and self-efficacy

Correlation analyses showed that physical self-esteem and self-efficacy were positively associated at the pretest, posttest, and change-score levels. These findings were consistent with H3. The largest association was observed between changes in physical self-esteem and changes in self-efficacy, suggesting that students who showed larger gains in physical self-esteem also tended to show larger gains in self-efficacy during the intervention. However, this association should be interpreted as covariation in psychological change rather than evidence of a causal or directional relationship.

This finding is consistent with the view that physical self-evaluations and efficacy beliefs are related but distinct psychological resources. Self-efficacy refers to individuals’ beliefs about their ability to organize and carry out actions needed to achieve desired outcomes, whereas physical self-worth and physical self-esteem reflect broader evaluations of the physical self (Bandura, 1997; Fox and Corbin, 1989). In a structured PE environment, these two psychological resources may change in parallel because students may receive both efficacy-related information and more positive feedback about their physical participation. For example, successful task completion, supportive peer feedback, and opportunities to contribute to group activities may be associated with both stronger beliefs that “I can do this” and a stronger sense that “my physical participation has value.” Nevertheless, these classroom processes were not directly measured in the present study, and therefore this interpretation should be treated as theoretically plausible rather than definitive.

The observed pattern is also consistent with prior research suggesting that student-centered learning strategies in PE can support autonomous motivation, psychological need satisfaction, effort, and PE learning-efficacy (Bechter et al., 2019), as well as adolescent PE research linking supportive motivational climates with more positive body image and physical self-perception (Jankauskienė et al., 2023). However, the present findings cannot determine whether changes in physical self-esteem contributed to changes in self-efficacy, whether the reverse occurred, or whether both changed in response to shared classroom experiences embedded in the SGTM instructional package.

Accordingly, these correlations are best understood as evidence of coordinated psychological change. This interpretation is compatible with longitudinal research showing reciprocal links between physical self-worth and physical activity (Raudsepp et al., 2013; Schmalz et al., 2007), but the present study cannot establish reciprocal causality. Future research should use multi-wave longitudinal designs, mediation models, cross-lagged approaches, or mixed-methods process data to clarify how physical self-esteem and self-efficacy may influence one another over time and through what classroom processes these coordinated changes may occur.

### 4.5 Theoretical and practical implications

The present study offers three implications for research on small-group instruction in PE. First, it moves the interpretation of small-group teaching beyond a general claim of instructional effectiveness by providing a more specific account of students’ psychological outcomes. The findings suggest that students in the SGTM condition showed more favorable posttest self-efficacy and physical self-esteem than students in the traditional-instruction condition after baseline adjustment. These constructs are important for adolescents’ motivation, body-related self-evaluations, and engagement in physical activity. However, because the present study did not directly assess the psychological mechanisms underlying these outcomes, the theoretical interpretation should be understood as preliminary and mechanism-oriented rather than confirmatory.

Second, the study provides a more nuanced account of physical self-perception outcomes. After Holm correction, physical self-worth was the only physical self-perception scale that retained a statistically significant group effect. This pattern is theoretically relevant because physical self-worth represents a more global evaluative component within the hierarchical structure of physical self-perception, whereas sport competence, physical condition, body attractiveness, and physical fitness represent more domain-specific perceptions. Thus, the findings suggest that classroom organization and peer-supported instructional structures may be particularly relevant to students’ broader evaluative relationship with their physical selves. However, because no direct statistical comparison of effect sizes across subscales was conducted, this pattern should not be interpreted as evidence that the effect on physical self-worth was statistically stronger than the effects on other physical self-perception domains.

Third, the study examined gender as a potential boundary condition without assuming that mean-level gender differences would necessarily translate into gender-differentiated intervention responses. The absence of statistically significant Group × Gender interactions suggests that clear gender differences in response to the SGTM condition were not detected in the present sample. However, this finding should be treated cautiously because the study had limited power to detect smaller interaction effects. Future research should examine whether gender-related differences emerge across different PE content areas, grouping strategies, teacher practices, and classroom climates.

From a practical perspective, the findings suggest that PE teachers may use small-group structures not only to increase participation, but also to create psychologically supportive learning environments. Effective implementation likely requires more than placing students into groups. Teachers may need to design tasks with attainable levels of challenge, use task cards to structure peer interaction, rotate leadership roles, encourage specific peer feedback, and reduce overly public forms of comparison. These practices may help students experience competence, receive social support, and develop a more positive sense of physical self-worth. At the same time, these implications should be understood as classroom-level possibilities associated with the implemented SGTM instructional package rather than broad policy recommendations or evidence that SGTM alone caused the observed outcomes. Future applications should also attend to teacher training and implementation fidelity, because the quality of task design, peer feedback, role rotation, and teacher guidance may influence whether small-group instruction produces supportive psychological experiences.

### 4.6 Limitations and future directions

Several limitations should be noted. First, the study was conducted with eighth-grade students from one middle school in Chengdu, China. Although students were individually randomized after baseline assessment, the participating school and teacher were selected by convenience, and the sample was limited in size and context. Therefore, selection bias and limited generalizability cannot be fully ruled out. Generalizability may also be constrained by cultural and contextual factors, including the Chinese PE curriculum context, school culture, class organization, teacher experience, and local expectations regarding PE participation. Future studies should include larger and more diverse samples from multiple schools and regions to test the generalizability of the findings.

Second, although the final sample provided acceptable sensitivity for detecting medium-to-large primary group differences, statistical power was limited for detecting smaller effects, particularly Group × Gender interactions and physical self-perception scale-level effects. Therefore, non-significant interaction and scale-level findings should be interpreted cautiously and should not be taken as definitive evidence of absence of effect. Future studies with larger samples are needed to more adequately examine whether gender moderates the psychological effects associated with SGTM and whether different physical self-perception domains respond differently to the intervention.

Third, biological maturation was not assessed in the present study. Given that participants were 13–14 years old, inter-individual differences in pubertal maturation may have influenced physical self-perceptions, self-esteem, self-efficacy, motor competence, and body image. Future research should consider including maturation-related indicators where feasible, such as self-reported pubertal status or other developmentally appropriate measures.

Fourth, the study relied on self-report measures of physical self-esteem and self-efficacy. Although the scales used in this study have prior validation support and questionnaire administration followed standardized procedures with anonymous responses, self-reported outcomes may still have been influenced by social desirability or perceived expectations. Future research could combine questionnaires with objective or externally rated indicators, such as classroom observations, behavioral engagement, motor performance records, teacher ratings, or peer interaction data. Such evidence would help clarify the classroom processes through which SGTM may shape students’ psychological experiences during PE lessons.

Finally, although using the same experienced PE teacher for both groups helped reduce between-teacher variability, teacher-specific characteristics may have influenced the implementation and outcomes. These characteristics may include teaching style, experience, enthusiasm, familiarity with students, and ability to implement structured peer interaction. Intervention implementation was supported through teacher training and classroom monitoring, but fidelity was not assessed using a standardized quantitative checklist or independent fidelity ratings. In addition, the study used a pretest–posttest design without long-term follow-up. Future studies should use multi-wave longitudinal designs, include multiple teachers and schools, apply formal fidelity assessments, and collect broader covariate measures to examine the durability of SGTM-related effects and the mechanisms through which they may occur.

## 5 Conclusion

This study examined the associations between a structured SGTM and junior high school students’ self-efficacy and physical self-esteem in PE. After controlling for baseline levels, students in the SGTM condition showed higher posttest self-efficacy and physical self-esteem than those in the traditional-instruction condition. Physical self-worth was the only physical self-perception scale that retained a statistically significant group effect after Holm correction.

These findings suggest that the implemented SGTM instructional package may be associated with more favorable psychological outcomes in PE, particularly students’ broader evaluative sense of their physical selves. However, the results should be interpreted cautiously because the psychological mechanisms were not directly assessed, and non-significant Group × Gender interactions should not be taken as definitive evidence that gender did not influence intervention responses. Future studies with larger samples, multiple teachers, formal fidelity assessments, longer follow-up, and process-based measures are needed to examine the durability, generalizability, and possible mechanisms of these outcomes.

## Data Availability

The raw data supporting the conclusions of this article will be made available by the authors, without undue reservation.
